# Cumulative Absorbed Dose and Successive Cyclic Reduction in Absorbed Dose Predict Response to ^177^Lu-DOTATATE in Neuroendocrine Tumors

**DOI:** 10.2967/jnumed.125.271039

**Published:** 2026-05

**Authors:** Mark J. Macsuka, Brandon Driscoll, Ivan W.T. Yeung, Julia Publicover, Ur Metser, Rosalyn Juergens, Sten D. Myrehaug, David Laidley, Rebecca K. Wong, Daniel R. McGowan, Katherine A. Vallis

**Affiliations:** 1 Department of Oncology, University of Oxford, Oxford, United Kingdom;; 2 Radiation Medicine Program, Princess Margaret Cancer Centre, University Health Network, Toronto, Ontario, Canada;; 3 Techna Institute, University Health Network, Toronto, Ontario, Canada;; 4 Southlake Regional Health Centre, Newmarket, Ontario, Canada;; 5 University Health Network, Toronto, Ontario, Canada;; 6 Joint Department of Medical Imaging, University Health Network, Toronto, Ontario, Canada;; 7 Division of Medical Oncology, Juravinski Caner Centre, Hamilton, Ontario, Canada;; 8 Department of Radiation Oncology, Odette Cancer Centre, Sunnybrook Health Sciences Centre, University of Toronto, Toronto, Ontario, Canada;; 9 University of Western Ontario, London, Ontario, Canada;; 10 Department of Radiation Oncology, University of Toronto, Toronto, Ontario, Canada; and; 11 Department of Medical Physics and Clinical Engineering, Oxford University Hospitals NHS Foundation Trust, Oxford, United Kingdom

**Keywords:** [^177^Lu]Lu-DOTATATE, neuroendocrine tumors, dosimetry, treatment response, radiopharmaceutical therapy

## Abstract

The aim of this study was to use image-derived dose metrics to predict the radiologic response of neuroendocrine tumors treated with [^177^Lu]Lu-DOTATATE. Particular focus was given to the evaluation of cyclic changes in absorbed dose per administered activity (AD/AA) as a potential prognostic factor. **Methods:** Data from 73 patients enrolled in the multicenter OZM-067 trial (NCT02743741) were analyzed. All patients who received 4 cycles of [^177^Lu]Lu-DOTATATE and underwent SPECT/CT imaging at 3 time points after each treatment were included. Tumor dosimetry was based on semiautomatic adaptive-threshold segmentations and recovery coefficient–based partial-volume correction; tumors smaller than 10 cm³ were excluded. If multiple tumors were segmented per patient, the mean absorbed dose (AD) and AD/AA were recorded at each cycle. Radiologic response was assessed using RECIST 1.1 criteria. **Results:** A significant decrease in AD/AA across cycles was observed, with a median decline of approximately 10% per cycle. Within this cohort, 28 patients had a partial response, 33 had stable disease, and 12 experienced disease progression. Responders exhibited a higher mean cumulative AD and greater decreases in AD/AA in successive cycles when compared with nonresponders. These metrics were uncorrelated predictors of response (*P* = 0.64). Notably, all 8 patients with an AD of at least 100 Gy and a decrease of at least 50% in AD/AA between cycles 1 and 4 were responders. Quantitative models combining AD and changes in AD/AA achieved an area under the receiver-operating-characteristic curve of 0.78. **Conclusion:** Both AD and changes in AD/AA were independently associated with radiologic response to [^177^Lu]Lu-DOTATATE in patients with neuroendocrine tumors. The consistent decrease in AD/AA over a course of therapy suggests a potential imaging biomarker that could inform adaptive treatment strategies, which should be further evaluated in a prospective setting and considered when designing dosimetry-guided [^177^Lu]Lu-DOTATATE trials.

## Visual Abstract

**Figure:**
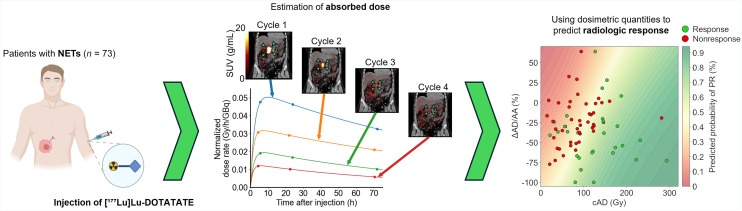

Radiopharmaceutical therapy (RPT) using [^177^Lu]Lu-DOTATATE is an established treatment modality for patients with somatostatin receptor 2–positive neuroendocrine tumors (NETs). Current treatment regimens, based on the NETTER trials, typically follow a fixed-activity protocol (1,2), delivering a constant administered activity (AA) across cycles. However, this one-size-fits-all approach overlooks opportunities for personalization, particularly when imaging and dosimetric data are available.

Absorbed dose (AD) delivered to tumors has long been considered a potential predictor of treatment response in RPT, and there is growing evidence of its association with clinical outcomes (3). However, efforts to establish a definitive dose–response relationship have led to heterogeneous results (4–8). One underappreciated factor is the dynamic change in tumor uptake across treatment cycles, as measured by the AD per AA (AD/AA), which has been shown to decrease in some NETs (4,7,9–13), neuroblastoma (14), and prostate tumors (15,16) during a course of RPT. Hohberg et al. have also reported that cyclic changes in AD/AA were different for responders and nonresponders in the context of [^177^Lu]Lu-PSMA RPT (15). A decrease in this metric could reflect biologic changes in tumors during therapy, such as loss of receptor expression or replacement of tumor cells by fibrotic tissue, both of which may signal treatment efficacy.

In this study, dosimetric data from the multicenter OZM-067 trial of [^177^Lu]Lu-DOTATATE therapy in patients with NETs were analyzed to determine whether AD and changes in AD/AA can be used to predict radiologic response.

## MATERIALS AND METHODS

### Treatment Administration

Eligible patients were enrolled in the prospective, multicenter, single-arm OZM-067 trial (NCT02743741). The primary endpoint of the main study was progression-free survival, as measured by RECIST 1.1, at 12-mo follow-up. The present substudy included patients who received all 4 cycles of [^177^Lu]Lu-DOTATATE and had SPECT images acquired at 4, 24, and 72 h after injection after each cycle. This exploratory post hoc analysis was approved by an institutional review board, and the need for written informed consent was waived.

In the main trial, the AA was varied to achieve a kidney AD of 23 Gy. At each cycle, the kidney AD and AD/AA were measured, and the AA required to deliver the remaining AD to reach 23 Gy was calculated and divided by the number of remaining cycles. The AA for the first cycle was standardized to 7.4 GBq, and the remaining AAs were capped at 11.1 GBq. AA reduction could also be implemented secondary to acute adverse events.

### Image Acquisition and Reconstruction

Imaging was performed using a Symbia Intevo (Siemens Healthineers), Optima 640 (GE HealthCare), or Infinia Hawkeye (GE HealthCare) SPECT/CT scanner using medium-energy general purpose collimators, acquiring 90 frames over 360° (30 s/frame). Images were reconstructed using Hybrid Recon Oncology version 4.0.6 (Hermes Medical Solutions AB) using a 20% photopeak centered at 208 keV. Downscatter was simulated during reconstruction, hence no scatter window data were used (17). Counts were converted to activity using calibration factors determined separately for each camera. Quantitative SPECT images were converted to AD rate maps using Voxel Dosimetry version 1.1.0 (Hermes Medical Solutions AB) (18). The specific settings are provided in the supplemental materials, available at http://jnm.snmjournals.org.

### Dosimetry

After generating AD rate maps, images were registered, volumes of interest were segmented, partial-volume correction was applied, a biexponential function was fitted to the AD rate estimates, and time integration was performed to arrive at the total AD for each cycle.

Each tumor was registered manually, using the 24-h scan as the reference. Registered scans were exported from Affinity workstation version 3.0.5 (Hermes Medical Solutions AB) for offline processing. Tumors were segmented on the 24-h scan by first enclosing them in a rough oversized volume that was later used as the starting point for contouring via a semiautomatic adaptive thresholding method implemented via a bespoke Python script (19). Tumors were included in this analysis if their volume was at least 10 cm^3^ when measured on the first-cycle SPECT using the adaptive thresholding segmentation method. Some patients had widespread diffuse disease, in the liver for example, and these were treated as a single tumor deposit. The final segmentation was copied to the other scans.

For the analysis of kidney AD, the 4- and 72-h scans were automatically registered to the 24-h scan via a translation operation determined by a gradient descent approach using a mean squares similarity metric that was implemented in SimpleITK (20). Segmentations of kidneys were automated using TotalSegmentator and corrected manually, if needed (21).

AD rates were corrected for the partial-volume effect, using recovery coefficients from phantom data (Supplemental Table 1). Partial-volume correction was applied to tumors and kidneys.

AD rate curves were fitted using a biexponential clearance model:

$$\dot{D}\left(t\right)=A\left(e^{-\lambda_{1}t}-e^{-\lambda_{2}t}\right),$$ Eq. 1

where $\dot{D}\left(t\right)$ is the AD rate as a function of time *t*, and *A*, $\lambda_{1}$, and $\lambda_{2}$ were fit parameters, with the latter 2 characterizing the washout and uptake rates. The washout half-time was constrained to be at least as fast as that of physical ^177^Lu decay (159.5 h). AD and AA were recorded for all treatment cycles. If a patient had multiple tumors that were eligible for inclusion in the dosimetry analysis, the mean AD and AD/AA were recorded.

Cyclic changes in AD/AA for tumors and kidneys were analyzed, with a focus on comparing the AD/AA of later cycles with that of the first cycle. The relationship between patient characteristics and cumulative tumor AD (cAD) or change in tumor AD/AA from cycle 1 to cycle 4 (ΔAD/AA) was also explored. Some information, particularly tumor grade, was established at the time of diagnosis, well before trial enrolment.

### Relationship Between Dose Metrics and Radiologic Response

Follow-up scans were scheduled every 4 mo for the first year and every 6 mo thereafter until 5 y after the end of therapy. At the time of this interim analysis, the best overall response was determined using imaging, which was available for up to 12 mo for all participants and up to 24 mo in some. Patients were classified as responders if their best response was complete response or partial response (PR), as defined by RECIST 1.1 (22). Other response categories included stable disease (SD) and progressive disease (PD). The relationship between these response categories and cAD or ΔAD/AA was then highlighted.

For groupwise analysis of dose–response relationships, patients were first sorted in ascending order of the dose metric of interest. Consecutive patients were then assigned to groups of 11, with the final group containing the remaining cases. For each group, the proportion of patients achieving PR was recorded, alongside the median dose metric within the group.

As part of the cAD–response analysis, the median cAD and proportion of patients achieving PR within each group was used to set up a best-fit model:

$$P_{\mathrm{PR}}\left(\mathrm{cAD}\right)=\;\frac{P_{\max}}{\left(1+e^{-\frac{\mathrm{cAD}-D_{\mathrm{half}}}{k}}\right)},$$ Eq. 2

where $P_{\mathrm{PR}}\left(\mathrm{cAD}\right)$ is the probability of PR as a function of cAD, $P_{\max}$ is the maximum probability of response observed (bounded between 0% and 100%), $D_{\mathrm{half}}$ is the cAD at which $P_{\mathrm{PR}}\left(\mathrm{cAD}\right)$ is half of the $P_{\max}$, and *k* is a scaling factor controlling the steepness of the curve. Goodness-of-fit for Equation 2 was quantified using $R^{2}$ (Eq. 3) and root-mean-square error (RMSE; Eq. 4):

R2=1−∑(PPR,observed−PPR,predicted)2 ∑(PPR,observed−∑PPR,observedn)2, Eq. 3

RMSE=∑(PPR,observed−PPR,predicted)2n, Eq. 4

where *n* is the number of groups.

Statistical models using both cAD and ΔAD/AA were fitted using 5-fold cross-validation with balanced groups. These models included 2-dimensional (2D) logistic regression and a support vector machine (SVM) classifier with a gaussian kernel. The 2D logistic regression model was defined as

PPRcAD,ΔAD/AA=11+e−β0+β1cAD+β2ΔAD/AA, Eq. 5

where $\beta_{0}$, $\beta_{1}$, and $\beta_{2}$ were coefficients to be estimated by minimizing a log-likelihood cost function. Separately, a SVM classifier was used to identify an optimal boundary separating responders from nonresponders in a 2D space, defined by cAD and ΔAD/AA, by maximizing the margin between the 2 classes, allowing for nonlinear relationships through kernel projection. Both 2D techniques were implemented using scikit-learn version 1.6.1 (23).

The prediction performance of all models was compared using the area under the receiver-operating-characteristic curve (ROC).

### Statistical Methods

Statistical significance was calculated using the Wilcoxon signed rank test for paired sample tests and the Wilcoxon rank sum statistic for independent sample tests. Correlation tests between continuous variables were quantified by the Spearman rank order coefficient. Statistical tests were performed in Python version 3.11.11, using SciPy version 1.13.0 (24). A *P* value of less than 0.05 was considered statistically significant.

## RESULTS

### Patient Characteristics

Of the 90 patients who completed therapy and all imaging sessions, 73 had at least 1 discrete lesion that was at least 10 cm^3^ in volume. In total, 137 such lesions were included in this analysis, 83 of which were located in the liver, 7 in the bone, and 47 in other organs. The mean number of tumors per patient was 1.9 (range, 1–6). Additional patient characteristics are shown in Table 1.

**TABLE 1.** Patient Characteristics (*n* = 73)

Characteristic	Value
*n*	73
Sex	
Male	49
Female	24
Age (y)	62 (32–83)
Body weight (kg)	81 (46–125)
Disease grade	
Grade 1	12
Grade 2	51
Grade 3	7
Unknown	3
Primary location	
Small bowel	32
Pancreas	18
Unknown	7
Colon	6
Lung	2
Adrenal	2
Stomach	2
Kidney	2
Paraganglioma	1
Ovary	1
AA per cycle (GBq)*	8.1 (1.7–12.7)
Cumulative AA (GBq)	37.1 (13.2–43.0)
Initial tumor volume (cm^3^)^†^	28.4 (10.0–2,340)

* AA was personalized on the basis of kidney uptake.

† Tumors smaller than 10 cm^3^ in volume were excluded.

Qualitative data expressed as number; continuous data expressed as median, followed by range in parentheses.

### Cyclic Changes of AD/AA

**FIGURE 1.**
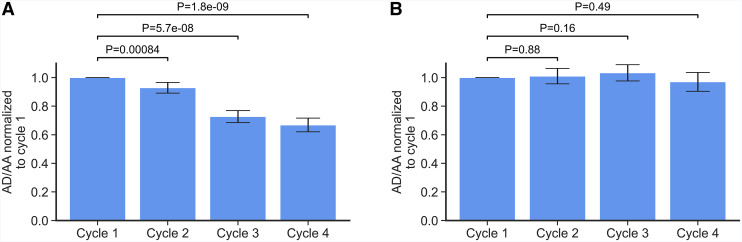
Average AD/AA significantly decreased for later cycles when compared with AD/AA of cycle 1 for tumors (A) but not for kidneys (B). Median values with SE are shown.

The median AD/AA values across all cycles are shown in Table 2. The percentage change of AD/AA at subsequent cycles compared with the baseline value at cycle 1 translated to significant reductions (7%, 27%, and 30% for cycles 2, 3, and 4, respectively) (Fig. 1). There was no significant difference between the magnitude of the decrease observed between successive cycles (Supplemental Fig. 1). No cyclic changes were observed in the kidneys (Fig. 1). In contrast to AD/AA, no significant changes in washout rate were observed (Table 2).

**TABLE 2.** Median Cyclic Changes of AD/AA and Washout Rates Across Treatment Cycles

	Cycle			
Variable	1	2	3	4
Tumor AD/AA (Gy/GBq)	3.2 ± 0.4	3.1 ± 0.3	2.4 ± 0.3	2.1 ± 0.2
Tumor washout rate (h)	79.5 ± 3.2	82.8 ± 3.8	79.2 ± 3.5	77.1 ± 3.6
Kidney AD/AA (Gy/GBq)	0.43 ± 0.03	0.42 ± 0.03	0.42 ± 0.04	0.42 ± 0.04
Kidney washout rate (h)	47.2 ± 2.1	48.1 ± 2.9	46.9 ± 2.7	46.8 ± 2.4

Data represent median ± SE.

### Relationship of cAD and ΔAD/AA to Patient Characteristics

**FIGURE 2.**
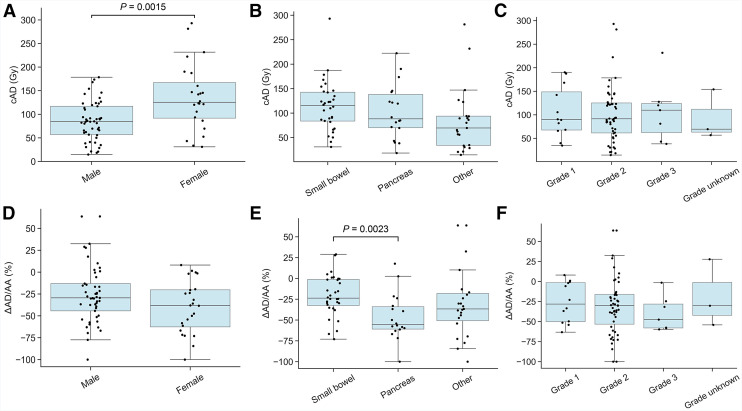
Relationship between cAD (A–C) and ΔAD/AA (D–F) with sex, primary location, and grade.

The distribution of cAD and ΔAD/AA when patients were stratified according to sex, tumor grade, and tumor primary location is shown in Figure 2. The ΔAD/AA was negative for most patients and significantly different from zero, regardless of stratification (Figs. 2D–2F). The cAD was significantly higher in women (Fig. 2A), whereas ΔAD/AA was significantly more negative for patients with pancreatic NETs compared with small-bowel NETs (Fig. 2E). No other significant relationships were observed.

### Differences of Dosimetric Quantities Between Responders and Nonresponders

At the time of this interim analysis, all 73 patients had completed imaging follow-up through 12 mo after therapy; 48 patients had additional assessments extending to 24 mo, and 25 had follow-up limited to 12 mo. PD, SD, and PR was observed in 12, 33, and 28 patients, respectively. Complete response was not observed in any patient.

**FIGURE 3.**
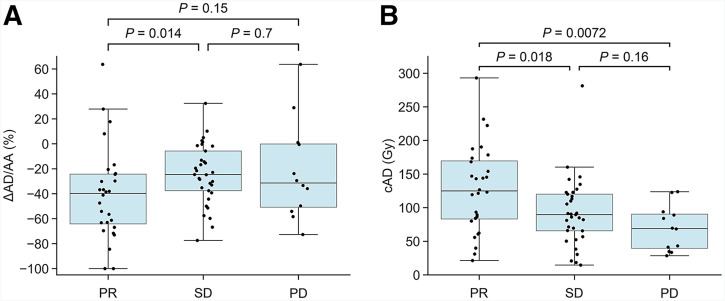
Relationship between ΔAD/AA (A) and cAD (B) and best overall radiologic response.

The relationships between radiologic response and cAD or ΔAD/AA were also investigated (Fig. 3). On average, patients with a PR had a higher tumor cAD and more pronounced decrease in AD/AA compared with patients with SD.

Because AA varied among cycles (Supplemental Fig. 2), it was tested as a confounding factor. No difference in cumulative AA was observed between responders and nonresponders, and no correlation was observed between cumulative AA and cAD (*P* = 0.97) or between the change in AA from cycles 1 to 4 and ΔAD/AA (*P* = 0.57) (Supplemental Fig. 3).

### Quantitative Analysis of the Relationships Among PR, cAD, and ΔAD/AA

**FIGURE 4.**
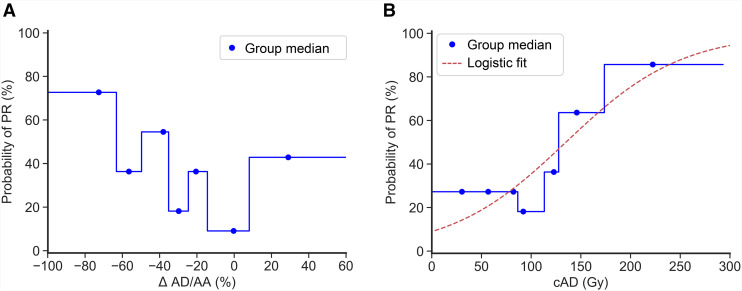
Probability of PR within patient subgroups defined by ΔAD/AA (A) or cAD (B).

As shown in Figure 4, an increase in the probability of response with a decrease in ΔAD/AA was observed. When patients were grouped by tumor cAD, a similar analysis revealed a steady positive relationship between cAD and response. Fitting Equation 2, this relationship was characterized by a $D_{\mathrm{half}\;}$of 135 Gy, *k* of 58 Gy, and $P_{\max}$ of 100%. *R*^2^ and RMSE goodness-of-fit metrics were 0.84 and 9.1%, and the area under the ROC curve with this model was 0.702.

**FIGURE 5.**
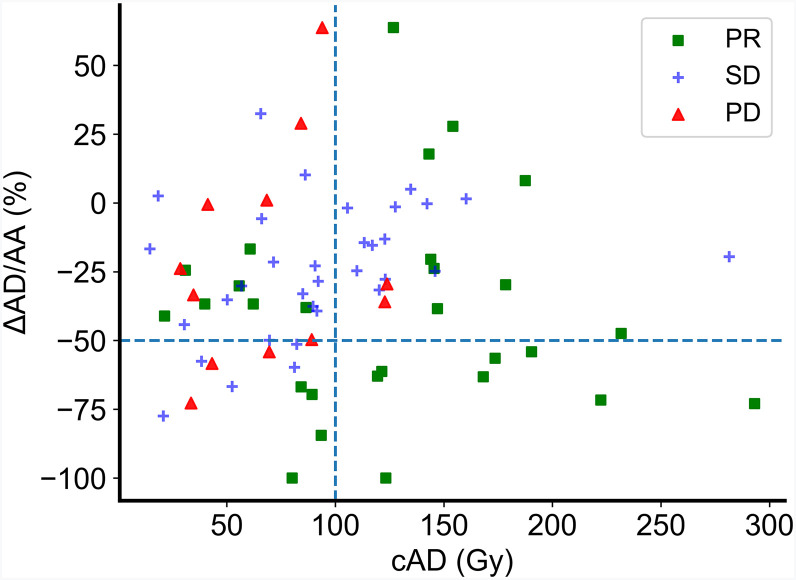
Relationships among cAD, ΔAD/AA, and radiologic response. Dashed lines represent arbitrary cutoffs of 100 Gy cAD and −50% ΔAD/AA.

No correlation was found between cAD and ΔAD/AA (*P* = 0.64), suggesting complementarity. Of 12 patients with PD, 10 had an average AD of less than 100 Gy; conversely, having a cAD of less than 100 Gy did not necessarily predict PD. Most patients with a cAD of at least 100 Gy had SD or a PR. Interestingly, all 8 patients with a cAD of at least 100 Gy and a ΔAD/AA of at least −50% had a PR (Fig. 5).

**FIGURE 6.**
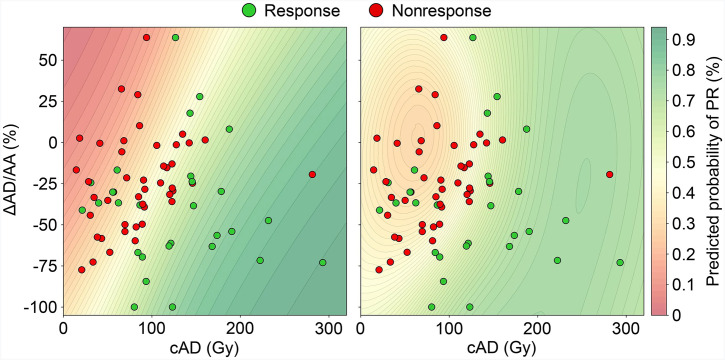
Predictive models of PR based on cAD and ΔAD/AA. Patients with SD and PD were merged into single nonresponder class for purposes of binary prediction of PR. Logistic regression is shown on left; SVM is shown on right.

The results of fitting 2D logistic regression and SVM models to the data in Figure 5 are shown in Figure 6. Both models were successful in predicting an increased probability of response for patients with a high cAD and large negative ΔAD/AA. The areas under the ROC curves were 0.714 and 0.781 for the logistic regression and SVM models, respectively. The ROC curves for all models are shown in Supplemental Figure 4.

## DISCUSSION

The aim of this study was to perform a quantitative analysis of image-derived dose metrics after [^177^Lu]Lu-DOTATATE therapy for NETs, with a focus on seeking biomarkers of radiologic response. If a patient had multiple tumors that were eligible for inclusion in the analysis, the mean AD and AD/AA were used, consistent with RECIST guidelines, which assign a single response metric, even in the case of metastatic disease. However, considerable intrapatient heterogeneity in the response of individual lesions to RPT is expected. We believe that patient-averaged metrics provide a pragmatic approach to personalized dosimetry that is simple to implement in clinical practice.

We found a significant absolute (Table 2) and relative (Fig. 1A) decrease in AD/AA between successive cycles, when compared with the first cycle. Combined with the fact that no such decrease in AD/AA in the kidneys was observed (Table 2; Fig. 1B), this provides further evidence that the current standard protocol of a constant AA for each cycle may be suboptimal, as the tumor-to-kidney uptake ratio decreases throughout the course of treatment.

As the washout rate remained unchanged between cycles (Table 2), the decrease in AD/AA arose from the loss of overall ability to accumulate the radioactivity in tumors. Cancer cell death in response to therapy is a possible explanation, but the lack of relationship between cAD and ΔAD/AA does not support this hypothesis as the only mechanism. It was further shown that the median decrease from any cycle to the next was on the order of 10%, with no significant differences between the magnitude of the reductions (Supplemental Fig. 1). This observation provides evidence that the mechanism behind the decrease in AD/AA did not plateau during the course of therapy.

The phenomenon of decreasing AD/AA has been reported by several investigators in the context of different cancers (4,7,9–12,14–16). In this cohort, the ΔAD/AA significantly differed from zero across all subgroups, indicating cyclic decreases in both pancreatic and small-bowel NETs, with a more pronounced effect in pancreatic NETs (Fig. 2). This contrasts with previous studies that reported no cyclic decline in small-bowel NETs in smaller cohorts (10,13). Our results suggest that cyclic reduction in uptake may be more generalizable than previously reported, although the magnitude may vary by primary tumor site. Additionally, we observed that cAD was significantly higher in women. Unlike the findings reported by Roth et al. (11), we found no difference in cAD or ΔAD/AA between tumors of different grades, possibly because grade information was recorded at the time of diagnosis, often years before patient enrollment.

Analysis revealed that patients with a PR had a high tumor cAD and large negative ΔAD/AA (Figs. 3 and 4). Differences in ΔAD/AA between responders and nonresponders have already been reported for [^177^Lu]Lu-PSMA RPT, suggesting that the mechanisms that underlie cyclic changes are not specific to cancer type or RPT (15). Changes in tumor vascularity in pancreatic NETs, determined using pretreatment and posttreatment contrast-enhanced CT scans, were reported in a clinical study (10), suggesting that an alteration in blood vessel density or function may contribute to this phenomenon. Tumors may also become fibrotic, as suggested by a comparison of tumor histology before and after neoadjuvant RPT in pancreatic NETs (25). Increasing tumor fibrosis, resulting in decreasing overall radiopharmaceutical uptake, has also been reported in preclinical studies (26).

As shown in Figure 4A, the probability of response gradually increased as the magnitude of ΔAD/AA increased, suggesting the possibility of using ΔAD/AA as an imaging biomarker of PR that precedes radiologic response. Interestingly, the group of patients with a positive ΔAD/AA seemed to have a higher proportion of responders compared with patient groups with a modest ΔAD/AA. This may have been the result of an overrepresentation of patients with a high average cAD (Fig. 4). More precisely, 43% of that group (3/7) received a tumor cAD of least 125 Gy. ADs of less than 100 Gy led to heterogeneous responses. Extending established radiobiologic models to RPT could be useful in exploring the intratumoral heterogeneity of dose deposition as a possible explanation. Radiobiologic modeling may reveal smaller-than-expected equivalent uniform doses, which may explain the lack of response at seemingly high average doses. Similarly, intrapatient lesional heterogeneity, as well as the heterogeneity of the patient cohort itself, could have affected our results.

cAD and ΔAD/AA were found to be uncorrelated, and it was possible to stratify patients on the basis of cAD and ΔAD/AA with more accuracy than with either metric alone, as measured by the areas under the ROC curves (Supplemental Fig. 4). Two machine-learning classifiers were used to provide a probability surface for quantitative predictions. Both models reinforced earlier insights that an increasing cAD and a negative ΔAD/AA led to higher probabilities of response in a synergistic manner, reaching predicted probabilities of greater than 90%.

It is reassuring that the SVM model led to conclusions similar to those of the more interpretable logistic regression model; however, data-driven approaches are reliable only when the quality of the data is high. Although every effort was made to provide accurate dosimetry, SPECT-based dosimetry inherently carries significant uncertainties, even when small tumors are excluded (as done in this study).

Combining the cAD and ΔAD/AA as presented here could pave the way for adaptive personalized therapeutic strategies, including adjusting the AA on the basis of optimal dose delivery by front-loading, or stopping treatment early once the probability of response is above a specific threshold. Although the AA varied among cycles (Supplemental Fig. 2), it was not related to response and was uncorrelated with cAD and ΔAD/AA (Supplemental Fig. 3). Therefore, the results presented here are expected to be generalizable to other administration schedules. However, as both the cAD and ΔAD/AA were calculated after the full course of therapy, a prospective study with adaptive AAs based on dose–response models is needed before clinical implementation. The findings detailed here can be used to predict radiologic response at the end of cycle 4, in contrast to the current convention, which assesses response using follow-up scans at intervals of 4–6 mo after treatment has concluded.

## CONCLUSION

Patient-averaged AD/AA decreased significantly across cycles and did so without reaching a saturation point during the course of therapy. Patients with a PR had a larger negative ΔAD/AA and higher cAD compared with nonresponders. Grouping patients by the average cAD or ΔAD/AA revealed that either metric could be used for response prediction. A dose–response curve was established that allowed for the modeling of expected response on the basis of the mean tumor dose per patient. As it was shown that cAD and ΔAD/AA were uncorrelated when predicting PR, the estimates in this study were improved by fitting multiparametric models that incorporated both metrics, which provided more accurate predictions. These results suggest that cAD and ΔAD/AA could be used simultaneously as imaging biomarkers to predict radiologic response to RPT at the conclusion of therapy as opposed to delayed follow-ups intervals. This contrasts with current practice, in which follow-up scans to assess response are often not performed until several months after the end of treatment.

## DISCLOSURE

This study was partially supported by GE HealthCare. Mark Macsuka is the recipient of an Oxford University Clarendon Scholarship. Daniel McGowan is supported by the Cancer Research U.K. National Cancer Imaging Translational Accelerator (C34326/A28684 and C42780/A27066). Katherine Vallis receives research funding from the MRC (UKRI805). No other potential conflict of interest relevant to this article was reported.

## Notes

KEY POINTS **QUESTION:** Can the cAD and ΔAD/AA over successive cycles predict radiologic response in patients undergoing [^177^Lu]Lu-DOTATATE therapy for NETs? **PERTINENT FINDINGS:** In this multicenter cohort of 73 patients receiving 4 cycles of [^177^Lu]Lu-DOTATATE with serial SPECT/CT dosimetry, both cAD and ΔAD/AA were associated with radiologic response. All patients with a cAD of at least 100 Gy and a decline of at least 50% in AD/AA achieved partial response. **IMPLICATIONS FOR PATIENT CARE:** AD and dynamic changes in AD/AA may serve as imaging biomarkers to predict radiologic response to [^177^Lu]Lu-DOTATATE therapy.
